# Supplementary material for: Rationalizing Graphene–ZnO Composites for Gas Sensing via Functionalization with Amines
Source: Nanomaterials (Basel). 2024 Apr 23;14(9):735. doi: 10.3390/nano14090735 (PMC11085583; doi:10.3390/nano14090735)
Supplement: Supplementary file 1 [file nanomaterials-14-00735-s001.zip › nanomaterials-2937292-supplementary.pdf]

Supporting Information for:

Rationalizing the formation of graphene-ZnO composites for gas sensing by applying graphene amination

Maxim K. Rabchinskii [1]*, Victor. V. Sysoev [2], Sergei A. Ryzhkov [1,2], Maksim A. Solomatin [2], Vladimir S. Gabrelian [1], Demid A. Kirilenko [1], Dina Yu. Stolyarova [3], Sviatoslav D. Saveliev [1,2], Alexander V. Shvidchenko [1], Polina D. Cherviakova [1], Alexey S. Varezhnikov [2], Sergei I. Pavlov [1], Maria Brzhezinskaya [4], Boris G. Khalturin [1], Nikita D. Prasolov [1], Pavel N. Brunkov [1]

[1] Ioffe Institute, Politekhnicheskaya St. 26, Saint Petersburg, 194021, Russia;

[2] Yuri Gagarin State Technical University of Saratov, Saratov 410054, Russia

[3] NRC "Kurchatov Institute", Akademika Kurchatova pl. 1, Moscow, 123182, Russia

[4] Helmholtz-Zentrum Berlin für Materialien und Energie, Hahn-Meitner-Platz 1, 14109 Berlin, Germany;

* - Dr. Maxim K. Rabchinskii, Ioffe Institute, Politekhnicheskaya St. 26, Saint Petersburg, 194021, Russia, e-mail: rabchinskii@mail.ioffe.ru

Section S1. Experimental setup for the gas-sensing studies

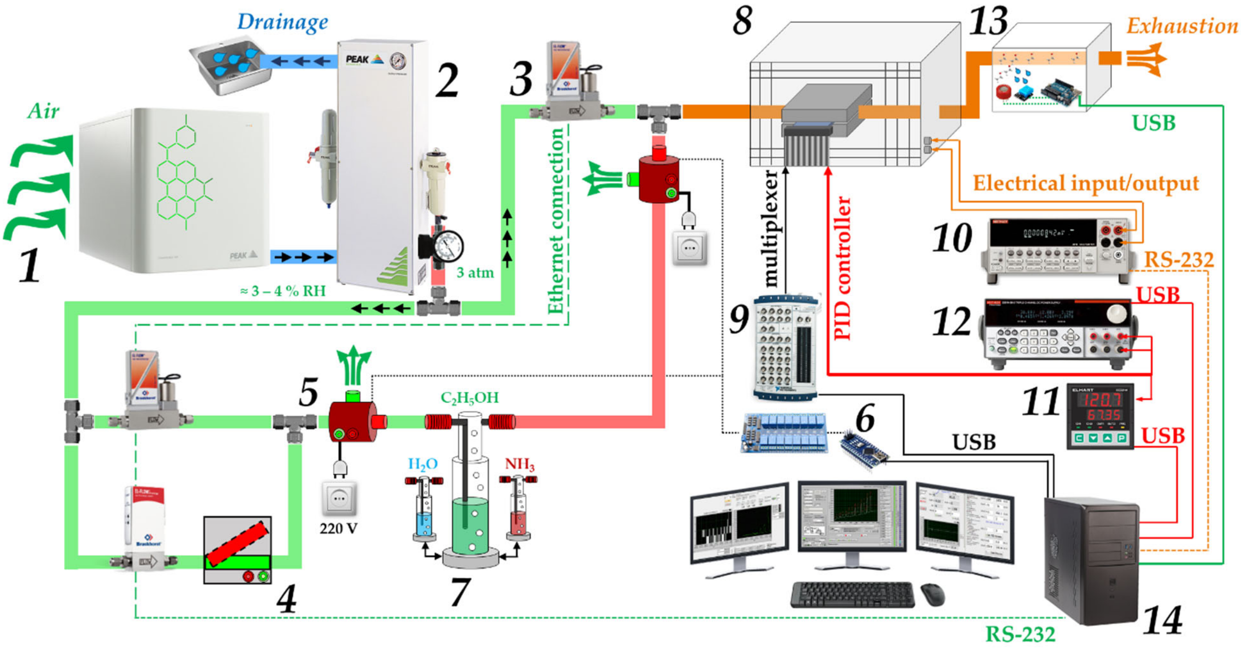

Figure S1. The scheme of the experimental setup to study the chemiresistive response of graphene-based chips: 1 – air compressor; 2 – filter dryer; 3 – precise mass-flow controller; 4 – two-way valve; 5 – three-way valve; 6 – relay, controlling the valves; 7 – bubblers, containing the analytes; 8 – Faraday cage, containing the chip mounted into a sealed stainless-steel chamber; 9 - data acquisition platform, NI-DAQ; 10 – multimeter (Keithley-2000); 11 – PID controller; 12 – power source for the heaters on the chip; 13 – exhaustion; 14 – PC with a home-made software to manage the setup.

S2. TEM characterization of the initial GO

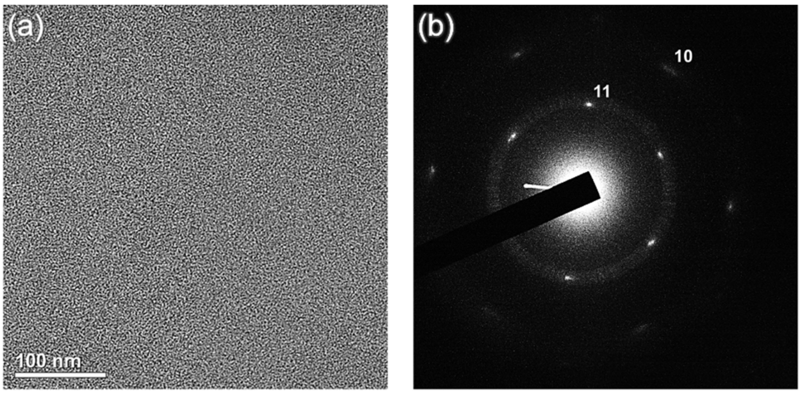

Figure S2. (a) TEM image and (b) the corresponding ED pattern of the initial graphene oxide

S3. Size distribution of the ZnO nanoparticles

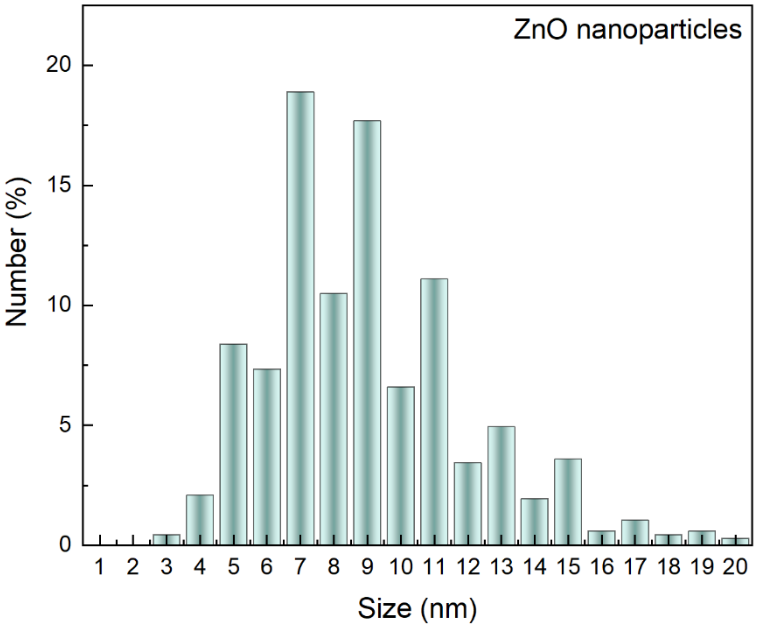

Figure S3. Size distribution of the ZnO nanoparticles derived from the collected TEM images

S4. TEM characterization of the initial rGO-ZnO composite

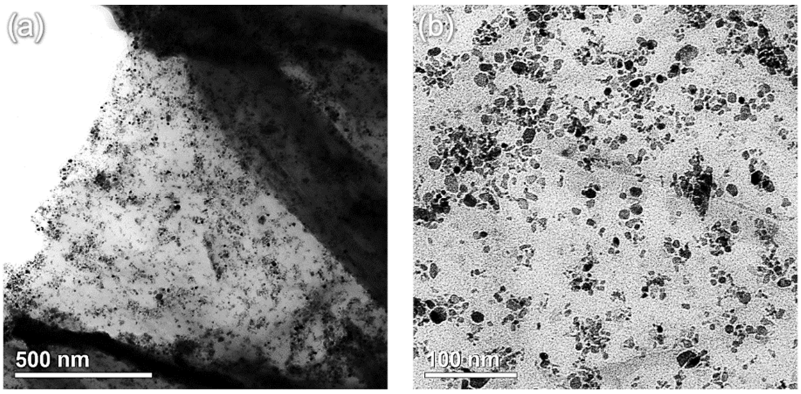

Figure S4. TEM images of the rGO-ZnO layer at different magnifications.

S5. TEM characterization of the initial rGO-ZnO composite after annealing

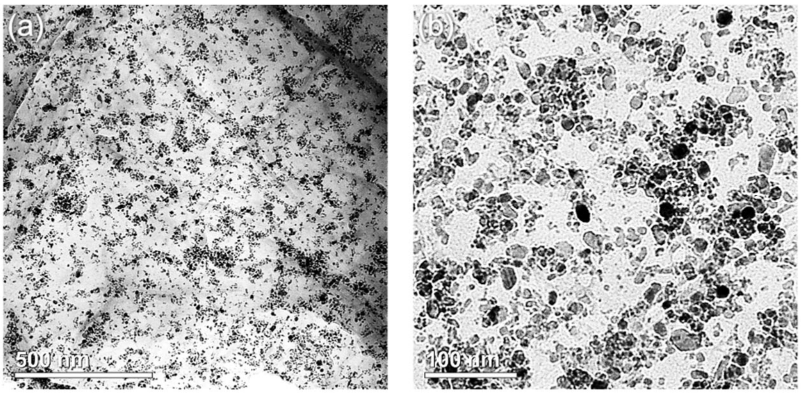

Figure S5. TEM images of the rGO-ZnO layer after annealing at different magnifications.

S6. Comparison of the On-chip multisensor array' performance towards the NH_3 and EtOH detection in dry air with the state-of-art graphene/carbon nanotubes-based gas sensing device

Table S1. Comparison of the gas sensors' performance towards the NH_3 detection

No.	Sensor type	Operating temperature	LoD	Recovery time	Selective detection of NH_3	Reference
1	On-chip multisensor array comprised of Am-ZnO	RT	5.1 ppm	7 min	Yes (LDA analysis)	This work
2	rGO-TiO_2	RT	~1 ppm	5 min	Yes	[R1]
3	Holey rGO	RT	<1 ppm	~1 min	Yes	[R2]
4	Carbon nanotubes network	RT	> 10 ppm	10 min	Yes	[R3]
5	Carboxylated graphene	RT	<1 ppm	>20 min	Yes (LDA analysis)	[R4]
6	Laser-synthesized graphene	RT	~10 ppm	3.5 min	Yes	[R5]
7	Fluorinated graphene	RT	~5 ppm	>4 min	Yes	[R6]
8	Phosphorus-doped graphene	RT	0.5 ppm	~ 2 min	Yes	[R7]
9	Carbonylated Graphene	RT	<1 ppm	20 min	Yes (LDA analysis)	[R8]

Table S2. Comparison of the gas sensors' performance towards the EtOH detection

No.	Sensor type	Operating temperature	LoD	Recovery time	Selective detection of EtOH	Reference
1	On-chip multisensor array comprised of Am-ZnO	RT	3.6 ppm	7 min	Yes (LDA analysis)	This work
2	GO with chemically diverse amine ligands	RT	25 ppm	1.5-7 min	No	[R9]
3	rGO-SnO_2 composite chemiresistor sensor	RT	3 ppm	2-3 min	No	[R10]
5	CoOEP-functionalized SWNT chemiresistor sensor	RT	~2 ppm	90 s	No	[R11]
6	Pt-activated SnO2 nanoparticles partly wrapped by (RGO)	110 °C	>100 ppm	20 s	No	[R12]
7	GO–aniline composite	RT	500 ppm	27 ms	No	[R13]
8	Inkjet-Printed rGO	RT	Saturated vapors	~ 20 min	No	[R14]
9	CVD-grown graphene nanoribbon films	150 °C	2 ppm	15 min	Yes (LDA analysis)	[R15]

References:

R1. Ye, Z.; Tai, H.; Guo, R.; Yuan, Z.; Liu, C.; Su, Y.; Chen, Z.; Jiang, Y. Excellent ammonia sensing performance of gas sensor based on graphene/titanium dioxide hybrid with improved morphology. *Appl. Surf. Sci.***2017**, *419*, 84–90. https://doi.org/10.1016/j.apsusc.2017.03.251.

R2. Yang, M.; Wang, Y.; Dong, L.; Xu, Z.; Liu, Y.; Hu, N.; Kong, E. S. W.; Zhao, J.; Peng, C. Gas Sensors Based on Chemically Reduced Holey Graphene Oxide Thin Films. *Nanoscale Res. Lett.* **2019**, *14*, 1–8. https://doi.org/10.1186/s11671-019-3060-5.

R3. Rigoni, F.; Freddi, S.; Pagliara, S.; Drera, G.; Sangaletti, L.; Suisse, J.-M.; Bouvet, M.; Malovichko, A. M.; Emelianov, A. V.; Bobrinetskiy, I. I. Humidity-enhanced sub-ppm sensitivity to ammonia of covalently functionalized single-wall carbon nanotube bundle layers. *Nanotechnology* **2017**, *28*, 255502. https://doi.org/10.1088/1361-6528/aa6da7.

R4. Rabchinskii, M. K.; Sysoev, V. V.; Glukhova, O. E.; Brzhezinskaya, M.; Stolyarova, D. Yu.; Varezhnikov, A. S.; Solomatin, M. A.; Barkov, P. V.; Kirilenko, D. A.; Pavlov, S.I. et al. Guding graphene Derivatization for the On-Chip Multisensor Arrays: From the Synthesis to the Theoretical Background. *Adv. Mater. Technol.* **2022**, *7*, 2101250. https://doi.org/10.1002/admt.202101250.

R5. Wu, D.; Peng, Q.; Wu, S.; Wang, G.; Deng, L.; Tai, H.; Wang, L.; Yang, Y.; Dong, L.; Zhao, Y.; et al. A Simple Graphene NH_3 Gas Sensor via Laser Direct Writing. *Sensors* **2018**, *18*, 4405. https://doi.org/10.3390/s18124405.

R6. Yuan, W.; Shi, G. Graphene-based gas sensors. *J. Mater. Chem. A* **2023**, *1*, 10078-10091. https://doi.org/10.1039/C3TA11774J.

R7. Niu, F.; Tao, L. M.; Deng, Y. C.; Wang, Q. H.; Song, W. G. Phosphorus doped graphene nanosheets for room temperature NH_3 sensing. *New J. Chem.*, **2014**, *38*, 2269-2272. https://doi.org/10.1039/C4NJ00162A.

R8. Rabchinskii, M. K.; Varezhnikov, A. S.; Sysoev, V. V.; Solomatin, M. A.; Ryzhkov, S. A.; Baidakova, M. V.; Stolyarova, D. Yu.; Shnitov, V. V.; Pavlov, S. I.; Kirilenko, D. A. et al. Hole-matrixed carbonylated graphene: synthesis, properties,

and highly-selective ammonia gas sensing. *Carbon* **2021**, *172*, 236-247. https://doi.org/10.1016/j.carbon.2020.09.087.
R9. Liu, B.; Huang, Y.; Kam, K. WL.; Cheung, W-.F.; Zhao, N.; Zheng, B. Functionalized Graphene-Based Chemiresistive Electronic Nose for Discrimination of Disease-Related Volatile Organic Compounds. *Biosens. Bioelectron.: X* **2019**, *1*, 100016. https://doi.org/10.1016/j.biosx.2019.100016.
R10. Pienutsa, N.; Roongruangsree, P.; Seedokbuab, V.; Yannawibut, K.; Phatoomvijitwong, C.; Srinives, S. Sno2-Graphene Composite Gas Sensor for a Room Temperature Detection of Ethanol. *Nanotechnology* **2021**, *32*, 115502. https://doi.org/10.1088/1361-6528/abcfea.
R11. Shirsat, S. M.; Bodkhe, G. A.; Sonawane, M. M.; Gawali, B. W.; Shirsat, M. D. Multivariate Analysis of a Cobalt Octaethyl Porphyrin-Functionalized SWNT Microsensor Device for Selective and Simultaneous Detection of Multiple Analytes. *J. Electron. Mater.* **2021**, *50*, 5780 – 5787. https://doi.org/10.1007/s11664-021-09111-3.
R12. Peng, R.; Chen, J.; Nie, X.; Li, D.; Si, P.; Feng, J.; Zhang, L.; Ci, L. Reduced Graphene Oxide Decorated Pt Activated SnO_2 Nanoparticles for Enhancing Methanol Sensing Performance. *J. Alloys Compd.* **2018**, *762*, 8-15. https://doi.org/10.1016/j.jallcom.2018.05.177.
R13. Zhu, X.; Zhang, J.; Xie, Q.; Hou, Z.-L. High-Sensitivity and Ultrafast-Response Ethanol Sensors Based on Graphene Oxide. *ACS Appl. Mater. Interfaces* **2020**, *12*, 38708–38713. https://doi.org/10.1021/acsami.0c12196.
R14. Dua, V.; Asurwade, S. P.; Ammu, S.; Agnihotra, S. R.; Jain, S.; Roberts, K. E.; Park, S.; Ruoff, R. S.; Manohar, S. K. All-Organic Vapor Sensor Using Inkjet-Printed Reduced Graphene Oxide. *Angew. Chem. Int. Ed.* **2010**, *49*, 2154. https://doi.org/10.1002/anie.200905089.
R15. Shekhirev, M.; Lipatov, A.; Torres, A.; Vorobeva, N.; Harkleroad, A.; Lashkov, A.; Sysoev, V.; Sinitskii, A. Highly Selective Gas Sensors Based on Graphene Nanoribbons Grown by Chemical Vapor Deposition. *ACS Appl. Mater. Interfaces* **2020**, *12*, 7392-7402. https://doi.org/10.1021/acsami.9b13946.
